# Physiological plasticity and local adaptation to elevated *p*CO_2_ in calcareous algae: an ontogenetic and geographic approach

**DOI:** 10.1111/eva.12411

**Published:** 2016-09-28

**Authors:** Jacqueline L. Padilla‐Gamiño, Juan Diego Gaitán‐Espitia, Morgan W. Kelly, Gretchen E. Hofmann

**Affiliations:** ^1^ School of Aquatic and Fishery SciencesUniversity of WashingtonSeattleWAUSA; ^2^ Department of BiologyCalifornia State University Dominguez HillsCarsonCAUSA; ^3^ Instituto de Ciencias Ambientales y EvolutivasFacultad de CienciasUniversidad Austral de ChileValdiviaChile; ^4^ CSIRO Oceans and AtmosphereHobartTASAustralia; ^5^ Department of Biological SciencesLouisiana State UniversityBaton RougeLAUSA; ^6^ Ecology, Evolution and Marine BiologyUniversity of California, Santa BarbaraSanta Barbara CAUSA

**Keywords:** California, life‐history stages, local adaptation, ocean acidification, photosynthesis, physiological plasticity, spore, upwelling

## Abstract

To project how ocean acidification will impact biological communities in the future, it is critical to understand the potential for local adaptation and the physiological plasticity of marine organisms throughout their entire life cycle, as some stages may be more vulnerable than others. Coralline algae are ecosystem engineers that play significant functional roles in oceans worldwide and are considered vulnerable to ocean acidification. Using different stages of coralline algae, we tested the hypothesis that populations living in environments with higher environmental variability and exposed to higher levels of pCO_2_ would be less affected by high pCO_2_ than populations from a more stable environment experiencing lower levels of pCO_2_. Our results show that spores are less sensitive to elevated pCO_2_ than adults. Spore growth and mortality were not affected by pCO_2_ level; however, elevated pCO_2_ negatively impacted the physiology and growth rates of adults, with stronger effects in populations that experienced both lower levels of pCO_2_ and lower variability in carbonate chemistry, suggesting local adaptation. Differences in physiological plasticity and the potential for adaptation could have important implications for the ecological and evolutionary responses of coralline algae to future environmental changes.

## 1 Introduction

From intertidal coasts to the bottom of the euphotic zone (Johansen, 1981; Steneck, 1986), coralline algae are major foundation species across marine ecosystems and around the world (Steneck & Dethier, 1994). These carbonate‐secreting organisms contribute to reef accretion (Adey, 1998; Chisholm, 2000) and CaCO_3_ production (Amado‐Filho et al., 2012), provide rigid substrate for organisms to settle (Daume, Brand‐Gardner, & Woelkerling, 1999; Gherardi & Bosence, 1999; Ritson‐Williams et al., 2009), and increase biodiversity by producing a more complex benthic topography (Nelson, 2009; Steller, Riosmena‐Rodriguez, Foster, & Roberts, 2003). Furthermore, coralline algae also host a great diversity of grazers and burrowing infauna (Chenelot, Jewett, & Hoberg, 2011) and produce secondary compounds that enhance settlement of invertebrates and trigger metamorphosis (Hay, 2009). Coralline algae in particular are considered vulnerable to the impacts of ocean acidification (Harley et al., 2012; Koch, Bowes, Ross, & Zhang, 2013; McCoy & Kamenos, 2015) because they form their skeletons from high Mg–calcite, the most soluble form of calcium carbonate (Borowitzka, Larkum, & Nockolds, 1974; Morse, Andersson, & Mackenzie, 2006). This study compared the responses of different life‐history stages of two populations of the articulate coralline algae *Corallina vancouveriensis* to different levels of pCO_2_. This alga is an abundant species along the intertidal coast of the California Current Large Marine Ecosystem (CCLME) which is one of the most productive and economically important ecosystems on Earth (Costanza et al., 1997). This ecosystem experiences high variability in water chemistry due to upwelling events and is particularly sensitive to ocean acidification and global warming (Gruber et al., 2012; Hauri et al., 2009).

Algal distribution is, in part, the result of adaptive responses to long‐ and short‐term fluctuations in the environment. Thus, understanding the degree of phenotypic flexibility and local adaptation is essential for predicting changes in their biogeographic distributions and to project future ecological trends in response to global changes. Furthermore, a complete ecophysiological understanding that includes multiple life‐history stages will help us to link physiological responses with fluctuations in the environment and to identify thresholds and vulnerabilities across the life cycle (Harley et al., 2012). Ocean acidification can impact physiological processes in algae such as photosynthesis, respiration, and growth, which are metabolically linked and can influence each other (Borowitzka et al., 1974; Gao et al., 1993; Martin, Charnoz, & Gattuso, 2013; Martin, Cohu, Vignot, Zimmerman, & Gattuso, 2013). CO_2_ enrichment can stimulate growth and photosynthesis by providing more substrate for carbon fixation; however, some species of algae have carbon concentration mechanisms (CCMs) that facilitate the acquisition of carbon from other sources (Giordano, Beardall, & Raven, 2005; Raven, Giordano, Beardall, & Maberly, 2012). In the genus Corallina, algae have evolved CCMs that allow them to transform ${\text{HCO}}_{3}^{-}$ (which is very abundant in the ocean) into CO_2_ and thus are not carbon‐limited. Species that do not possess CCMs are generally carbon‐limited under current concentration of seawater CO_2_ and thus are more likely to respond positively to elevated pCO_2_ (Kubler, Johnston, & Raven, 1999). Thus, algal responses to pCO_2_ will depend, in part, on the availability of carbon sources and the mechanisms present to obtain them.

The physiological response of calcifying algae to ocean acidification is highly variable, most likely reflecting the high diversity in this group, variation in photosynthetic pathways and calcification mechanisms, and variation in acclimatization capacity of different species (Koch et al., 2013). Previous studies have found that increased overall pCO_2_ availability can enhance photosynthetic rates but decrease calcification and enhance dissolution in calcifying algae (Koch et al., 2013; Semesi, Kangwe, & Björk, 2009). Lower calcification rates under high pCO_2_ concentrations were observed in *Corallina pilulifera*,* C. sessilis,* and *C. officinalis* (Gao & Zheng, 2010; Gao et al., 1993; Hofmann, Yildiz, Hanelt, & Bischof, 2012). Lower growth and reduced photosynthesis in response to high pCO_2_ concentrations were observed in *C. officinalis* and *C. sessilis* (Gao & Zheng, 2010; Hofmann et al., 2012), and for the latter, the negative effects of CO_2_ were enhanced when algae were exposed to UVR (Gao & Zheng, 2010). Similarly, elevated temperatures and nutrients can enhance the negative effects of ocean acidification in calcifying algae (Anthony, Kline, Diaz‐Pulido, Dove, & Hoegh‐Guldberg, 2008; Diaz‐Pulido, Anthony, Kline, Dove, & Hoegh‐Guldberg, 2012; Johnson & Carpenter, 2012; Martin & Gattuso, 2009; Russell, Thompson, Falkenberg, & Connell, 2009; Sinutok, Hill, Doblin, Wuhrer, & Ralph, 2011). Ocean acidification can also weaken the structural integrity of coralline algae (Ragazzola et al., 2012) and cause tissue damage (Martin & Gattuso, 2009), which reduces their ability to resist wave energy and boring by predators. Ecological interactions of coralline algae with other coralline algae, non‐calcified algae and/or grazers can also be affected by ocean acidification (Johnson & Carpenter, 2012; Jokiel et al., 2008; Kroeker, Micheli, & Gambi, 2012; Kuffner, Andersson, Jokiel, Rodgers, & Mackenzie, 2007; McCoy & Pfister, 2014; Porzio, Buia, & Hall‐Spencer, 2011).

Currently, relatively little is known about the effects of ocean acidification on early life‐history stages of coralline algae (Bradassi, Cumani, Bressan, & Dupont, 2013; Cumani, Bradassi, Di Pascoli, & Bressan, 2010; Kroeker et al., 2012; Kuffner et al., 2007; Roleda et al., 2015) or about the capacity for local adaptation in this important group. Only two studies have looked at spore development of crustose coralline algae under ocean acidification (Bradassi et al., 2013; Cumani et al., 2010), and to our knowledge, only one study has looked at the effect of ocean acidification on spore development in articulate coralline algae (Roleda et al., 2015). *Corallina vancouveriensis* reproduce by releasing spores (Johansen, 1981), which can fully attach to the bottom within hours of release (Miklasz, 2012) and recruit near the parental alga. The capacity of coralline algae to attach rapidly could limit dispersal distance, restrict gene flow among populations, and increase the potential for local adaptation in this species (Endler, 1977). Local adaptation can produce differences in physiology and life history and provide advantages in fitness in the local environment. Distinguishing spatial patterns of local adaptation and the relative contribution of local adaptation and phenotypic plasticity to organismal performance will help us to understand and predict the impacts of climate change and implement effective practices to manage marine ecosystems.

To explore the role of local adaptation and whether differences in physiological responses to high pCO_2_ are consistent with regional differences in carbonate chemistry patterns, we cultured spores and adults of *C. vancouveriensis* from different populations. By measuring survival, growth, photosynthesis, and other physiological parameters, we explored the tolerance of different life stages to high pCO_2_, and whether different populations were locally adapted to environments with different pCO_2_ levels. We hypothesized that populations of *C. vancouveriensis* living in environments with higher environmental variability (due to upwelling) and exposed to higher levels of pCO_2_ would be less affected by similar high pCO_2_ levels than populations from more stable environments experiencing lower pCO_2_ levels.

## 2 Methods

### 2.1 Algal collections and sensor deployment

*Corallina vancouveriensis* Yendo (1902) is a common articulate coralline algae in the CCLME. It is light pink to light purple in color and can form dense mats on emergent bedrock or in tidepools in mid‐to‐low intertidal zones of exposed habitats. Specimens of *C. vancouveriensis* were collected during low tides at four sites spanning Point Conception in Central California. North of Point Conception, they were collected at Cambria (35.665°N, 121.276°W) and Arroyo Grande (35.528°N, 121.078°W); south of Point Conception, they were collected at Santa Barbara (34.407°N, 119.874°W) and Carpinteria (34.388°N, 119.517°W). These locations correspond to two regions with different oceanographic conditions (Cudaback, 2005). Sites north of Point Conception experience high wave exposure and waters are around 3–4°C colder due to higher recurrence of coastal upwelling (Blanchette, Miner, & Gaines, 2002), whereas south of Point Conception waters are warmer and the shore is more protected from heavy wave action (O'Reilly & Guza, 1993).

Algae were individually stored in plastic bags, placed in a cooler, and transported to the University of California Santa Barbara. In the laboratory, algal fronds were thoroughly and gently cleaned of epiphytic organisms and accumulated sediments and placed in tanks with running seawater at 14–15°C. Healthy algal specimens, that is, without alteration of the cortical tissue or discoloration, were selected to obtain spores and perform physiological experiments. Algal collections occurred at two different times: February 2013 for spore experiments and April 2013 for adult experiments.

Temperature and pH sensors were deployed north and south of Point Conception in 2013, as a part of a larger network of sensors making continuous measurements at sites throughout the CCLME (Hofmann et al., 2014, Chan et al. *in prep*). Temperature and pH were measured every 20 min using Durafet^®^‐based (Honeywell Inc.) pH sensors that were custom‐designed for near‐shore deployment (Chan et al. *in prep*). Sensors were secured to the bedrock and placed submerged in tide pools in the lower part of the intertidal zone. Unfortunately, sensors at our collection sites failed to record pCO_2_. Thus, pCO_2_ data were obtained from the closest network sensors to our collection sites (34°28.03N, 120°16.69W north of Point Conception and 34°43.14N, 120°36.53 south of Point Conception). Spearman correlation analyses were conducted to compare the environments (temperature) at the sites where the sensors were located and the sites where the algal collections were performed.

### 2.2 Algal culturing and seawater chemistry

Adults and spores of *C. vancouveriensis* were cultured under different pCO_2_ levels using a flow‐through CO_2_ mixing system as described in Fangue et al. (2010). The system blends dry, CO_2_‐free atmospheric air with pure CO_2_ to produce different pCO_2_ levels using mass flow controllers. Gas for each mixture was continually delivered to gas‐mixing reservoirs for equilibration with seawater to achieve a desired pCO_2_ level. One header tank was used for each pCO_2_ treatment, and each treatment had two experimental tanks that were randomized with interdependent replicates within treatments (Cornwall & Hurd, 2015). CO_2_‐equilibrated seawater was then transferred from the reservoir buckets to the larval buckets for the duration of the experiment using lawn irrigation drippers. The system was modified by replacing culture buckets with rectangular tanks and adding LED lights overhead (MarineLand Reef). Small submersible aquarium pumps (Aquatop, 70 gph) and pipes were used to provide uniform water flow inside the tanks, with a flow rate ~1 cm/s. Two pCO_2_ levels were compared: for adults ~410 μatm (pH = 8.0) and 1,033 μatm (pH = 7.7); for spores ~485 (pH = 8.0) and 1,186 μatm (pH = 7.6) (Table 1). Temperature, salinity, and pH were measured daily for each pCO_2_ experimental treatment according to best‐practice procedures (Dickson, Sabine, & Christian, 2007; Fangue et al., 2010). Temperature was measured using a wire thermocouple (Themolyne PM 207000/Series 1218), and salinity was measured using a conductivity meter (YSI 3100). pH was determined following the standard operating procedure (SOP) 6b (Dickson et al., 2007) using a spectrophotometer (Bio Spec‐1601; Shimadzu) and dye m‐cresol purple (Sigma‐Aldrich) as the indicator. Total alkalinity (TA) was measured every 3 days in the reservoir buckets, following the SOP 3b (Dickson et al., 2007). Water samples for TA were collected using borosilicate glass‐stoppered bottles, poisoned with mercuric chloride, and analyzed at a later time using a potentiometric titration procedure with a commercially available titration unit (T50; Mettler Toledo) and following the SOP 3b (Dickson et al., 2007). Both pH and alkalinity were assessed for accuracy using certified reference materials (CRMs) from Dickson (Scripps Institution of Oceanography), Batch 8 (pH = 8.0923 + 0.0004) and Batch 103 (TA = 2232.94 + 0.79 mmol/kg) for pH and alkalinity, respectively. Parameters of pCO_2_, Ω_ara_, and Ω_cal_ were calculated using CO_2_calc (Robbins, Hansen, Kleypass, & S. Meylan, 2010) with the dissociation constants of Mehrbach, Culberson, Hawley, and Pytkowicz (1973). The irradiance levels for the experimental treatments were set to 30.5 ± 2.4 μmol photon m^−2^ s^−1^ (*SE*) under a 12‐hr light: 12‐hr dark photoperiod using LED lights (MarineLand Reef). Irradiance was measured as photosynthetic active radiation using MKV‐L spherical sensors (Alec Electronics, Kobe, Japan). Irradiance levels were set to ~30 μmol because levels >40 −mol have been suggested to lead to tissue death in indoor cultures (Gao et al., 1993). Temperature, salinity, and carbonate parameters of seawater used in experimental treatments are shown in Table 1.

**Table 1:** Temperature, salinity, and seawater carbonate chemistry parameters of seawater used in experimental treatments for spores and adults of the algae *Corallina vancouveriensis* (mean and *SE*)

Life stage	Treatment	Parameter	Tank 1	Tank 2	Average
Spores	Low CO_2_	pCO_2_ (μatm)	492.3 ± 46.4	477.6 ± 45.4	485.0
		pH	8 ± 0.0	8.0 ± 0.0	8.0
		Temp. (°C)	15.3 ± 0.7	15.0 ± 0.3	15.1
		Salinity (psu)	33.2 ± 0.1	33.2 ± 0.1	33.2
		TA (μmol/kg SW)	2222.3 ± 7.1	2222.2 ± 7.1	2222.3
		Ca	3.2 ± 0.3	3.2 ± 0.3	3.2
		Ar	2.1 ± 0.2	2.1 ± 0.2	2.1
	High CO_2_	pCO_2_ (μatm)	1177.0 ± 66.1	1195.2 ± 47.5	1186.1
		pH	7.6 ± 0.0	7.6 ± 0.0	7.6
		Temp. (°C)	15.1 ± 0.2	15.0 ± 0.3	15.0
		Salinity (psu)	33.2 ± 0.1	33.2 ± 0.1	33.2
		TA (μmol/kg SW)	2224.8 ± 5.6	2224.8 ± 5.6	2224.8
		Ca	1.5 ± 0.1	1.5 ± 0.1	1.5
		Ar	1.0 ± 0.0	1.0 ± 0.0	1.0
Adults	Low CO_2_	pCO_2_ (μatm)	398.9 ± 153.4	421.9 ± 143.8	410.4
		pH	8.0 ± 0.1	8.0 ± 0.1	8.0
		Temp. (°C)	14.1 ± 0.3	14.2 ± 0.3	14.1
		Salinity (psu)	33.2 ± 0.3	33.2 ± 0.3	33.2
		TA (μmol/kg SW)	1961.3 ± 691.3	2027.5 ± 610.0	1994.4
		Ca	2.9 ± 1.0	3.0 ± 0.9	2.9
		Ar	1.9 ± 0.7	1.9 ± 0.6	1.9
	High CO_2_	pCO_2_ (μatm)	1018.6 ± 111.0	1048.1 ± 102.0	1033.4
		pH	7.7 ± 0.0	7.7 ± 0.0	7.7
		Temp. (°C)	13.9 ± 0.2	13.9 ± 0.2	13.9
		Salinity (psu)	33.2 ± 0.3	33.2 ± 0.3	33.2
		TA (μmol/kg SW)	2224.4 ± 7.9	2224.4 ± 7.9	2224.4
		Ca	1.7 ± 0.1	1.6 ± 0.1	1.6
		Ar	1.1 ± 0.1	1.0 ± 0.1	1.0

### 2.3 Spore and crust (juvenile) growth and mortality

Within 3 days after collection, a subset of algal specimens was haphazardly selected to obtain spores. Five or six fronds (~6–7 cm) per individual were placed on previously labeled cover glass slides in a 300‐ml container filled with filtered seawater. Lids were placed on containers, and fronds were left to release spores naturally for 1 day at room temperature (19–20°C). After 24 hr, the cover glass slides with spores were transferred to the experimental tanks (high and low pCO_2_ treatments in duplicate) and cultured for 19 days. Each experimental unit received slides with spores from the four different sites (*n* = 15–17 for Santa Barbara, *n* = 14–16 for Carpinteria, *n* = 5–7 for Cambria, and *n* = 8–10 for Arroyo Grande). The number of slides per site was dependent on the amount of spore material released by the algae: *n* = 19, 14, 8, 10 (high pCO_2_ tank 1); *n* = 18, 14, 5, 8 (high pCO_2_ tank 2); *n* = 23, 16, 7, 7 (low pCO_2_ tank 1); *n* = 15, 14, 7, 11 (low pCO_2_ tank 2), Santa Barbara, Carpinteria, Cambria, and Arroyo Grande, respectively. Spore growth and mortality were monitored and recorded under both low and high pCO_2_ conditions at 3 and 19 days after settlement using a dissecting scope and a digital camera (Jenoptik). Growth rates were estimated by measuring crust surface area over time using ImageJ software ver. 1.42 (Abramoff, Magalhaes, & Ram, 2004). Photographs were taken under 8× magnification, using a grid under the glass slide to ensure that the same spores were photographed every time.

### 2.4 Adult physiology and growth

From each site, 12 adult algae (*n* = 48) were selected for physiological analyses. Six young branches (~1.5 cm and 100–120 mg fresh weight) were excised from each individual and randomly assigned to the experimental treatments. These branches were inserted upright into plastic grids at the bottom of experimental tanks and cultured for 30 days at 14 ± 1°C under both low (~410 μatm) and high pCO_2_ (~1030 μatm) conditions (Table 1).

#### 2.4.1 Metabolic rates and primary productivity

Net primary productivity (NPP) and respiration (R) were measured at the beginning (Day 0) and at the end (Day 30) of the experimental treatments using the light and dark bottle methodology described in Howarth and Michaels (2000). These physiological rates were assessed as changes in dissolved oxygen concentrations during light and dark incubations, respectively. In brief, branches were placed in 50‐ml acrylic chambers filled with seawater at the same pCO_2_ as the experimental tank that was previously filtered and sterilized with UV light. Metabolic chambers were kept in a temperature‐ and light‐controlled incubation tanks (30 ± 2.5 μmol photon m^−2^ s^−1^ and 14 ± 1°C) for 3 hr in order to avoid oxygen saturation greater than 120% during light incubation and to maintain oxygen saturation above 80% at the end of the dark incubation (Noisette, Egilsdottir, Davoult, & Martin, 2013). Periodically, acrylic chambers were gently agitated to break up the boundary layer surrounding the algae. Initial and final dissolved O_2_ concentrations were measured using a fiber optic O_2_ sensor probe (Foxy‐R; Ocean Optics, Dunedin, FL, USA) attached to a fluorescence‐based optical sensor (NeoFox^®^; Ocean Optics) and connected to a computer running the manufacturer’s software (NeoFox Viewer). The sensor was calibrated with a zero solution (sodium sulfite and 0.01 M sodium tetraborate) and air‐saturated seawater (100%). Calibration points were measured once the O_2_ signal equalized and remained constant (~10 min). No blank corrections were applied because oxygen values in light and dark control chambers remained constant. Dry weight of each branch was measured after drying the sample for 48 hr at 68°C to normalize metabolic and photosynthetic rates following (Egilsdottir, Noisette, Noël, Olafsson, & Martin, 2012).

#### 2.4.2 Growth rates and biochemical components

Vegetative growth of each algae branch was determined by changes in the wet weight between the beginning and the end of the experiment. The relative growth rate (RGR), expressed as percentage increase in fresh weight biomass per day (%/day), was estimated assuming exponential growth during the culture period according to the formula: RGR = 100 × ( ln *W*_*t*_ − ln *W*_0_)/*t*, where *W*_0_ represents the initial and *W*_*t*_ the final wet weight of the algae, and *t* is the time of culture in days.

Photosynthetic pigments were measured following Gao and Zheng (2010). About 0.1 g FW per sample was ground and placed in 10 ml absolute methanol at 4°C in darkness for 24 hr. Chlorophyll *a* and carotenoids were determined spectrophotometrically according to Wellburn (1994). For phycobiliproteins (i.e. phycocyanin and phycoerythrin), samples of about 0.1 g FW were placed in 5 ml of 0.1 M phosphate buffer (pH 6.8), ground at 4°C, and rinsed with a further 5 ml of buffer for 24 hr. The concentrations of phycobiliproteins were measured spectrophotometrically using the chromatic equations of (Beer & Eshel, 1985). All pigments were measured using the spectrophotometer Bio Spec‐1601 (Shimadzu) after centrifugation at 5,000 g for 15 min.

### 2.5 Statistical analyses

Crust (juvenile) growth data were square‐root‐transformed and analyzed using a general linear model with site and pCO_2_ level as fixed factors and tank nested within pCO_2_ treatment. Spore mortality data were analyzed using a generalized linear mixed model (GLMM; binomial distribution) with site and pCO_2_ level as fixed factors and tanks and algae ID as random factors. Tank was nested within pCO_2_ treatment. These analyses were run via the glmer function of the R‐package lme4. Tukey's honestly significant difference multiple comparison tests were conducted as post hoc tests when GLMMs detected significant differences using the R‐package “LMERConvenienceFunctions.”

Adult physiological measurements (NPP, gross primary productivity [GPP], and respiration) and biochemical components (pigments) were analyzed using robust two‐way repeated‐measures ANOVA with the R‐package “WRS2.” A general linear model was used to analyze growth at each site, time, and pCO_2_ level. Tukey's honestly significant difference multiple comparison tests were conducted as post hoc tests when GLMs detected significant differences. Normality and homogeneity were tested using the quantile–quantile plot (QQPlot) and the hovPlot() function in the HH package, respectively.

## 3 Results

**Figure 1:**
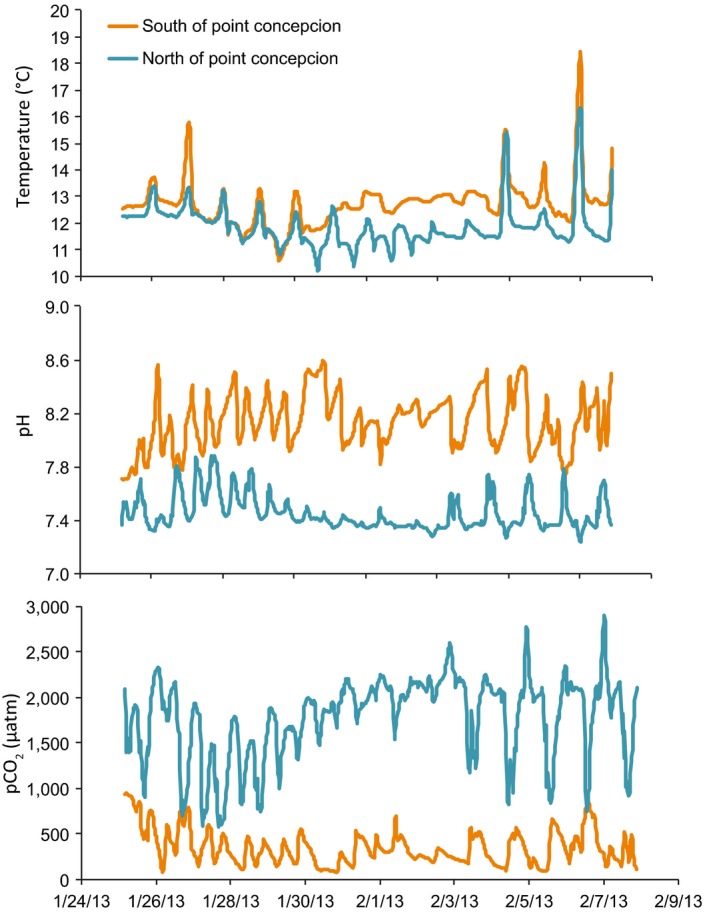
Temperature, pH, and dissolved pCO_2_ at two intertidal sites located north and south of Point Conception in the California Current Large Marine Ecosystem

We collected algae from four sites, which consistently experience different strengths and durations of upwelling events (Blanchette et al., 2002). Sensors deployed in winter 2013 revealed strong differences in temperature and carbonate chemistry between intertidal sites located north and south of Point Conception (Fig. 1). At the northern site, pCO_2_ reached a maximum of 2,904 μatm (pH = 7.24) and varied around a mean pH of 7.45 ± 0.13 and a mean pCO_2_ of 1,770 ± 450 μatm, whereas at the southern site, pCO_2_ reached a maximum of 946.6 μatm (pH = 7.7) and varied around a mean pH of 8.14 ± 0.2 and a mean pCO_2_ of 350 ± 189 μatm. Correlations of temperature time series showed a strong positive relationship between collections sites and the sensor sites both north and south of Point Conception (Spearman correlation coefficient for northern sites ρ = .705, *p* < .001 and southern sites ρ = .631, *p* < .001, Fig. S1).

### 3.1 Spore growth and mortality

**Figure 2:**
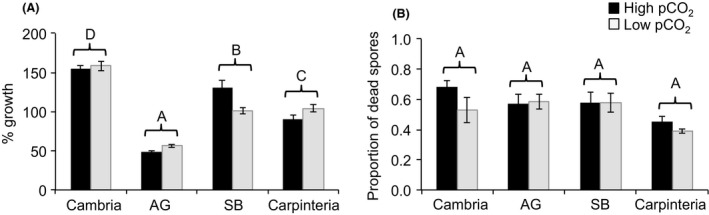
Percent growth rate (a) and mortality (b) of spores of *Corallina vancouveriensis* under high and low pCO_2_ levels. North of Point Conception: Cambria and AG, Arroyo Grande and south of Point Conception: SB, Santa Barbara and Carpinteria (*n* = 15–17 for SB,* n* = 14–16 for Carpinteria, *n* = 5–7 for Cambria, and *n* = 8–10 for AG, mean ± *SE*). Shared letters indicate means that were not significantly different after a post hoc comparison

Crust growth differed significantly among the four sites (Fig. 2a, Table 2); crusts from Cambria showed the largest surface area at the end of the experiment (156.85% increase after 16 days), followed by Carpinteria and Santa Barbara with growth increases of 116.02% and 97.32%, respectively. Arroyo Grande crusts had the smallest surface areas at the end of the experiment (only 52.25% increase). We found that spore growth in response to high pCO_2_ differed among populations, with higher growth rates in Santa Barbara compared to the other populations (Tukey's multiple comparison, *p* < .05; Fig. 2a, Table 2). Although crusts from Arroyo Grande, Cambria, and Carpinteria showed slightly lower growth under high pCO_2_ (Fig. 2a), our Tukey's comparison test revealed that these differences were not statistically significant. Mortality of spores differed among sites (Table 2, Fig. 2) but did not differ between pCO_2_ treatments (Fig. 2b, Table 2). These differences were explained by the lowest proportion of dead spores in Carpinteria (southern population) compared to the other sites (Tukey's multiple comparison, *p* < .05).

**Table 2:** Effects of pCO_2_ and local environment on the physiology of the algae *Corallina vancouveriensis*

Stage	Parameter	Tank (CO_2_)		CO_2_		Site		pCO_2_ × site	
		*F*	*p*	*F*	*p*	*F*	*p*	*F*	*p*
Spores	Growth	0.84	.43	0.02	.893	40.14	**<.001**	3.43	**.018**
	Mortality	1.86	.18	0.57	.449	5.35	**.001**	0.83	.475
Adults at the beginning of experiment	NPP	1.23	.29	61.05	**<.001**	19.87	**<.001**	16.36	**<.001**
	GPP	1.4	.25	50.55	**<.001**	16.39	**<.001**	12.99	**<.001**
	Respiration	1.57	.21	0.29	.593	0.12	.949	0.12	.489
Adults at the end of experiment	NPP (T30)	0.77	.46	366.00	**<.001**	1.06	.368	0.81	.489
	GPP	0.61	.54	278.00	**<.001**	0.63	.597	0.34	.798
	Respiration	0.66	.52	243.00	**<.001**	4.58	**.004**	3.26	**.025**
	Growth	0.77	.46	609.46	**<.001**	8.14	**<.001**	8.78	**<.001**
	Chlorophyll a	1.69	.19	32.40	**<.001**	0.14	.937	0.17	.914
	Carotenoids	0.8	.045	0.00	.949	0.08	.971	0.12	.947
	Phycocyanin	0.93	.4	55.60	**<.001**	0.24	.867	0.07	.978
	Phycoerythrin	0.55	.58	60.57	**<.001**	0.32	.807	0.10	.959

Significant values at 95 % confidence (p < .05) are in bold.

### 3.2 Adult physiology and growth

**Figure 3:**
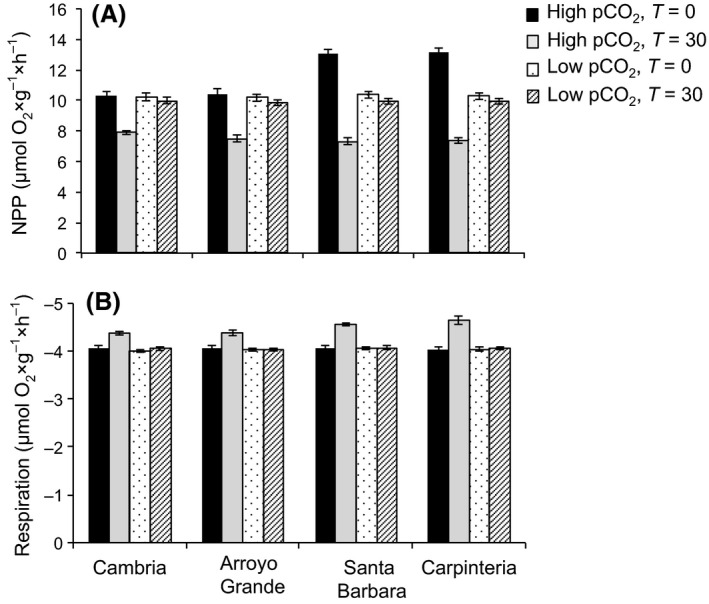
Net photosynthetic productivity (A) and respiration (B) under high and low pCO_2_ levels in adults of *Corallina vancouveriensis* from sites in California located north (Cambria and Arroyo Grande) and south (Santa Barbara and Carpinteria) of Point Conception (*n* = 24, mean ± *SE*). Solid bars represent treatments under high pCO_2_ at the beginning of the experiment (black) and after 30 days (gray). Bars with dots and strips represent treatments under low pCO_2_ at the beginning of the experiment (dots) and after 30 days (strips)

Net primary productivity, GPP, and respiration rates of *C. vancouveriensis* differed greatly depending on pCO_2_ level and the population of origin (Figs 3, S2, Table 2). Under low pCO_2_ (~400 μatm), algae from all sites had similar NPP, GPP, and respiration rates (*p* > .05, Figs 3, S2). However, algae from southern sites (Santa Barbara & Carpinteria) had higher NPP and GPP rates than algae from the northern sites when initially exposed to higher levels of pCO_2_ (~1,030 μatm) (*F*_3,88_ = 16.36, *p* < .001 and Tukey's multiple comparison, *p* < .001, Figs 3, S2). In the low pCO_2_ NPP, GPP and respiration did not change after culturing the algae for 30 days (*p* > .05, Figs 3, S2). However, NPP and GPP at all sites decreased significantly after 30 days of exposure to high pCO_2_ levels (*F*_3,88_ = 60.07 and 41.35, respectively, *p* < .001, Figs 3, S2). At the northern sites, the decrease in NPP was approximately 20%, whereas at the southern sites, the decrease in NPP was around 46% (*F*_3,88_ = 51.98, *p* < .001, and Tukey's multiple comparison, *p* < .001, Fig. 3). GPP showed very similar trends, 17% and 30% decrease at northern and southern sites, respectively (*F*_3,88_ = 35.07, *p* < .001 and Tukey's multiple comparison, *p* < .001, Fig. S1). In contrast to photosynthetic rates, respiratory rates increased for all sites after culturing the algae at high pCO_2_ levels for 30 days (*F*_3,88_ = 3.5, *p* = .018, and Tukey's multiple comparison, *p* < .001, Fig. 3), while respiration remained unchanged in the low pCO_2_ treatment after 30 days (*p* > .05, Fig. 3).

**Figure 4:**
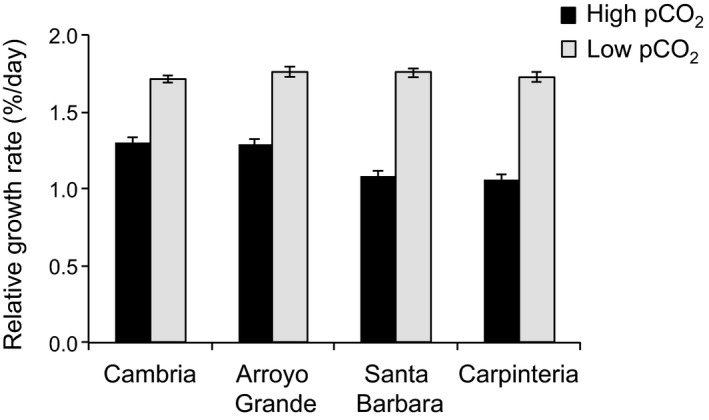
Relative growth rate in adults of *Corallina vancouveriensis* exposed to high and low pCO_2_ levels. Adults were collected from sites in California located north (Cambria and Arroyo Grande) and south (Santa Barbara and Carpinteria) of Point Conception (*n* = 24, mean ± *SE*)

**Figure 5:**
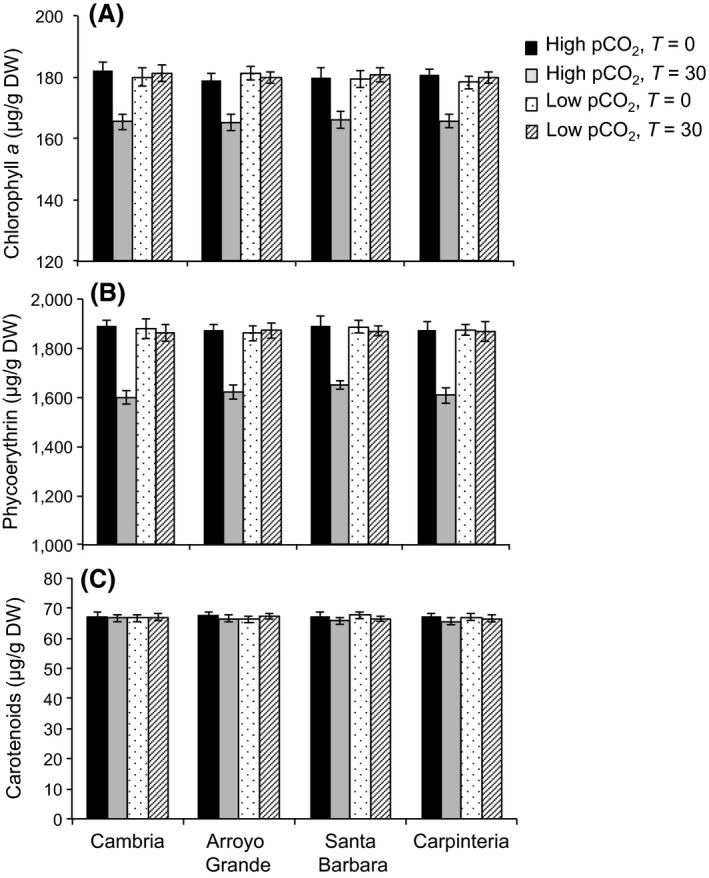
Pigment content in adults of *Corallina vancouveriensis* exposed to high and low pCO_2_ levels. Adults were collected from sites in California located north (Cambria and Arroyo Grande) and south (Santa Barbara and Carpinteria) of Point Conception (*n* = 12, mean ± *SE*). Solid bars represent treatments under high pCO_2_ at the beginning of the experiment (black) and after 30 days (gray). Bars with dots and strips represent treatments under low pCO_2_ at the beginning of the experiment (dots) and after 30 days (strips)

Relative growth rates of *C. vancouveriensis* showed a significant interaction between pCO_2_ treatment and site (*F*_3,184_ = 8.78, *p* < .001, Fig. 4). Overall, RGR did not differ between populations under low pCO_2_ (Tukey's multiple comparison, *p* > .05, Fig. 4) but decreased by 24% and 35% under high pCO_2_ in northern and southern populations, respectively (Tukey's multiple comparison, *p* < .001, Fig. 4). Different responses were found between pigments in response to high pCO_2_ levels. Chlorophyll *a*, phycocyanin, and phycoerythrin decreased after culturing for 30 days under high pCO_2_ (*F*_1,88_ = 66.19, Figs 5, S2), and this response was consistent among populations (Tukey's multiple comparison, *p* < .001). Carotenoids, however, did not change between populations or pCO_2_ levels (*p* > .05, Fig. 5). Data available at the Dryad Digital Repository: http://dx.doi.org/10.5061/dryad.8jn67.

## 4 Discussion

Selection pressures can differ greatly between environments and life stages and contribute to the variability in physiological plasticity and the potential for adaptation to future environmental changes. The goal of this study was to compare the performance of different life stages of the alga *C. vancouveriensis* under different levels of pCO_2_. Our results demonstrate that the response of *C. vancouveriensis* to pCO_2_ depends on population, life stage, and the duration of exposure to pCO_2_. Adults from sites experiencing lower and less variable pCO_2_ levels (southern sites) were more sensitive to the highest tested pCO_2_. Before acclimation to the different pCO_2_ levels, algae showed higher photosynthesis under high pCO_2_ than adults from the northern site. However, after 30 days of exposure to high pCO_2_, adults from all sites showed a reduction in photosynthesis and growth. Nevertheless, adults from the northern site (higher upwelling) experienced a smaller decrease in growth in response to high pCO_2_. This can be explained by the fact that the tested high pCO_2_ level is outside the natural range of environmental variability for the southern population. In contrast, populations at the northern site frequently experience pCO_2_ levels higher than 1,100 μatm (Fig. 1). The differences in response of each population may be attributable to phenotypic plasticity (given the different environmental histories of the adults before collection) or to the effects of natural selection (Harley et al., 2012). Some have argued that within the natural range of pCO_2_ variability, plasticity will play the major role in alleviating the effects of high pCO_2_, while outside the natural range, evolutionary and/or transgenerational effects may be more relevant (Calosi et al., 2013; Thor & Dupont, 2015). Only a few studies have tested for evolutionary adaptation to natural variation in pCO_2_ (Kelly, Padilla‐Gamiño, & Hofmann, 2013); however, the thermal tolerance literature abounds with examples of adaptive differences in thermal optima between populations, even when the tested temperatures do not fall outside the natural range of variability (Angilletta, Niewiarowski, & Navas, 2002; Sanford & Kelly, 2011). Therefore, it seems equally possible that the differences observed between populations in this study represent adaptive differences shaped by average differences in average pH, rather than the extremes. Using a broader range of partial pressures of pCO_2_ will help to investigate whether there is a tipping point in the growth and calcification of *C. vancouveriensis* from the northern populations and how it relates to the high pCO_2_ variability at those locations. In other taxa such as mussels and sea urchins, tipping points in larval physiology have been associated with the extreme pCO_2_ values of the present natural variability (Dorey, Lançon, Thorndyke, & Dupont, 2013; Ventura, Schulz, & Dupont, 2016). However, not all organisms show a clear threshold in physiological decline (i.e., tipping point) in response to pCO_2_ values above extreme values in the natural environment (Comeau, Edmunds, Spindel, & Carpenter, 2013). Moreover, future studies using a broader range of pCO_2_ levels should use one header tank per experimental unit to achieve true replication (Cornwall & Hurd, 2015). In our design, we had experimental units with interdependent treatment replicates (Design B4, Cornwall & Hurd, 2015), which may confound the effect of treatment with inherent differences between tanks.

Reduced growth and/or calcification under high pCO_2_ levels has also been observed in adults of the calcifying algal species *C. pilulifera*,* Hydrolithon* sp., *Halimeda incrassata*,* Neogoniolithon* sp., *Lithotamnion corallioides*,* Arthrocardia corymbosa*, and *Porolithon onkodes* (Anthony et al., 2008; Cornwall et al., 2013; Gao & Zheng, 2010; Gao et al., 1993; Johnson & Carpenter, 2012; Noisette et al., 2013; Ries, Cohen, & McCorkle, 2009; Semesi et al., 2009). However, elevated pCO_2_ did not have an effect on growth in the intertidal coralline algae *Corallina elongata* (Egilsdottir et al., 2012), and in *Litophyllum cabiochae,* increased pCO_2_ even enhanced calcification (Martin, Charnoz, et al., 2013; Martin, Cohu, et al., 2013). Lower growth rates in response to high pCO_2_ may be a result of decreased calcification due to lower photosynthetic rates and higher respiration and/or increased dissolution associated with a lower saturation state. As the pCO_2_ increases and saturation state decreases, it becomes more energetically costly to calcify. Our high pCO_2_ treatments were nearly undersaturated with respect to calcite and aragonite, with Ω_ara_ and Ω_cal_ values close or at the saturation horizon (Ω = 1) (Kleypas, 1999). Differences in carbonate mineralogy could also explain the variation in growth responses among species; corallinales have high variability in mineralogical composition and can change their skeletal mineralogy in response to the local seawater chemistry (i.e., Mg^2+^ concentrations) (Smith, Sutherland, Kregting, Farr, & Winter, 2012). As Mg–calcite is the most soluble form of CaCO_3_, higher Mg–calcite content in the skeleton can increase dissolution and reduce resistance to elevated pCO_2_ (Morse et al., 2006).

Despite the fact that pCO_2_ enrichment can stimulate photosynthesis by providing more substrate for carbon fixation, we observed lower photosynthetic rates (NPP and GPP) in response to high pCO_2_ in *C. vancouveriensis*. This result is consistent with previous studies examining photosynthetic responses of coralline algae to high CO_2_ levels (Anthony et al., 2008; Gao & Zheng, 2010; Gao et al., 1993; Martin, Charnoz, et al., 2013; Martin, Cohu, et al., 2013). Nevertheless, other studies have found enhanced photosynthesis under high pCO_2_ (Semesi et al., 2009) or no effects at all (Egilsdottir et al., 2012; Hofmann et al., 2012; Semesi et al., 2009). Photosynthesis in the Corallina genus is not strictly carbon‐limited; they have evolved processes such as the CCMs that allow them to increase the amount of CO_2_ around Rubisco (enzyme involved in fixing CO_2_ during photosynthesis) via ion channels or by catalyzing the transformation of ${\text{HCO}}_{3}^{-}$ into CO_2_ (Giordano et al., 2005; Raven, Beardall, & Giordano, 2014; Raven et al., 2012). Differences within and between algal species in response to high pCO_2_ may be due to the presence and activity of these CCM (Giordano et al., 2005) and whether they involve external or intracellular carbonic anhydrase (Reinfelder, 2011); an enzyme that catalyzes the interconversion of dissolved bicarbonates and carbon dioxide.

Furthermore, it has been shown that in coralline algae, the primary carbon used in photosynthesis is ${\text{HCO}}_{3}^{-}$ (Borowitzka, 1981), and in *C. pilulifera,* calcification and photosynthesis can be enhanced by carbonate (${\text{CO}}_{3}^{2-}$) and bicarbonate (${\text{HCO}}_{3}^{-}$) but not by the addition of free CO_2_ (Gao et al., 1993). In the coralline algae, *C. officinalis* carbonic anhydrase (CA) activity was ~40% higher in individuals grown under high pCO_2_ than individuals grown in ambient conditions for 4 weeks (Hofmann et al. 2012) contrary to the expectation that CA would be downregulated when more pCO_2_ was available. However, after a long‐term exposure (12 weeks), *C. officinalis* showed an inverse trend between CA activity and pCO_2_ concentration. Future studies are needed to better understand how CO_2_ can be regulated and concentrated at the Rubisco fixation site and how elevated CO_2_ will impact the activity of CA and the operation and interactions of CCM (Koch et al., 2013).

Respiration rates of *C. vancouveriensis* from all tested populations increased after culturing them under high pCO_2_ for 30 days, indicating greater physiological demands for algae growing under this treatment. Our results are consistent with Noisette et al. (2013), who found increased respiration, lower calcification, and higher occurrence of bleaching in response to high pCO_2_ in the intertidal coralline alga *Lithophyllum incrustans*. These studies suggest that high pCO_2_ increases metabolic demands and that poor physiological state could affect the calcification balance and increase susceptibility to other stressors (Martin, Charnoz, et al., 2013; Martin, Cohu, et al., 2013; Noisette et al., 2013), possibly compromising long‐term survival and reproduction. Conversely, other studies on coralline algae did not find effects of high pCO_2_ on respiration (Egilsdottir et al., 2012; Hofmann et al., 2012; Johnson, Moriarty, & Carpenter, 2014; Martin, Charnoz, et al., 2013; Martin, Cohu, et al., 2013; Noisette et al., 2013; Semesi et al., 2009). Higher respiratory rates under high pCO_2_ in our study were associated with lower net and gross photosynthetic rates and lower chlorophyll and phycobiliprotein content. Chl *a*, phycocyanin, and phycoerythrin decreased ~8%, 14% and 11%, respectively, under high pCO_2_, indicating a reduction in photosynthetic potential (capacity to absorb light) for the four populations studied after the 30‐day acclimation. Interestingly, *L. incrustans* did not show differences in photosynthesis or chlorophyll content between pCO_2_ levels (Noisette et al., 2013). Carotenoids, which also absorb light energy for photosynthesis and protect the chlorophyll from photodamage, were not affected by high pCO_2_. Similar results for carotenoids under high pCO_2_ were found in *C. sessilis* and the diatom *Phaeodactylum tricornutum* (Gao & Zheng, 2010; Li, Gao, Villafañe, & Helbling, 2012).

To understand how physiological responses can impact individual and population responses to ocean acidification, we also need to consider the different thresholds and limits of different life stages, not just the adults. Currently, little is known about the effects of ocean acidification on early life‐history stages of coralline algae, despite the fact that these stages can be very susceptible to high levels of pCO_2_ (Bradassi et al., 2013; Cumani et al., 2010; Kroeker et al., 2012; Kuffner et al., 2007; Roleda et al., 2015) and possibly act as demographic bottleneck for benthic recruitment under acidified conditions.

In California, adults of *C. vancouveriensis* release spores throughout the year with no seasonal trends (Miklasz, 2012). Spores of coralline algae are not calcified; they are negatively buoyant and attach to the substratum using developing filaments that attach to surface microstructures (Steneck, 1986), or using mucilage and epoxy‐like resins that can harden over time (Fletcher & Callow, 1992). Spores of *C. vancouveriensis* can fully attach after 24 hrs of settlement, but some spores can achieve attachment within 1 hr of release (Miklasz, 2012). Once they have attached in the substratum, they flatten, and calcification starts after the first cell division (Walker and Moss 1984). Germination can occur in as little as 8 hr (Miklasz, 2012) suggesting that *C. vancouveriensis* has limited dispersal and a high potential for local adaptation (Hereford, 2009; Leimu & Fischer, 2008).

Our results show that early life‐history stages of *C. vancouveriensis* were more resilient to the direct effects of near‐future acidification levels than adults. However, future work will need to be performed to eliminate the possibility that these differences are due to seasonality, because experiments with adults were performed around 2 months earlier than experiments with spores.

Spore growth and survival did not differ between pCO_2_ treatments; however, spores at each site had different growth rates. Interestingly, growth rates did not differ consistently between regions; the largest and smallest growth rates were found in algae from sites at the northern region (150% and 50% growth, Cambria and Arroyo Grande, respectively), whereas algae from the southern region had more similar growth rates (116% and 97% growth, Santa Barbara and Carpinteria, respectively). Differences in growth between sites may be due to maternal effects or variability in growth requirements between sites. Likewise, Bradassi et al. (2013) found no differences in growth in early life stages of the CCA *Phymatolithon lenormandii*, but observed increased mortality, abnormal thalli, and different calcification patterns (margin of the thallus vs. total area of the thallus) under high pCO_2_ levels. Negative impacts of high pCO_2_ were also seen in early life stages of the crustose coralline *L. incrustans* including low spore production and growth and increased mortality of the germination disks (Cumani et al., 2010). Furthermore, in recruits of *A. corymbosa* (articulate coralline algae), lower growth rates and decrease in Mg–calcite content were observed under low pH treatments (Roleda et al., 2015). However, these responses were not as pronounced as in the adults of the same species (Cornwall et al., 2013) suggesting that juveniles may be more resistant than adults to lower pH. In mesocosm experiments, Kuffner et al. (2007) found that recruitment of tropical crustose coralline algae decreased 78% with elevated seawater carbon dioxide concentration, whereas Kroeker et al. (2012) found no effects of low pH in the recruitment of temperate coralline algae settling in plates located at a volcanic CO_2_ site. These contrasting results highlight the variability in sensitivity of early stages of different species in response to elevated pCO_2_ and the limitations to projecting individual and population‐level responses to ocean acidification without considering variation in tolerances of different life‐history stages within a species (Harley et al., 2012). However, Kuffner et al. (2007) used diluted hydrochloric acid to reduce pH of the water in the experimental treatments, which could lead to different outcomes than if CO_2_ was used to acidify the water. It is also important to note that differences in physiological response between populations may not only be attributed to differences in pCO_2_ natural conditions between sites but also to the simultaneous exposure of other parameters associated with upwelling events such as low temperatures and high nutrient concentrations. Future experimental studies manipulating pCO_2_ to simulate long‐term environmental variability should be performed to conclusively differentiate the effects of pCO_2_ between populations.

Our work highlights the importance of considering complete life cycles when projecting the biological impacts of future environmental changes, because different stages will have different physiological thresholds and tolerance limits. Our study suggests that spores are less sensitive to high pCO_2_ than adults of C*. vancouveriensis*. The physiology and growth rates of adults were impacted at the highest pCO_2_. The tested scenarios were only relevant in the context of ocean acidification for the southern sites experiencing lower upwelling, and individuals from these populations were more sensitive to the high pCO_2_. Adults from the northern populations are already experiencing high pCO_2_ tested in this study and as predicted were more tolerant to these levels.

## Data Archiving Statement

Data for this study are available at the Dryad Digital Repository: http://dx.doi.org/10.5061/dryad.8jn67.
