# Supplementary figures and images for: Physiological plasticity and local adaptation to elevated pCO 2 in calcareous algae: an ontogenetic and geographic approach
Source: Evol Appl. 2016 Sep 28;9(9):1043–53. doi: 10.1111/eva.12411 (PMC5039319; doi:10.1111/eva.12411)

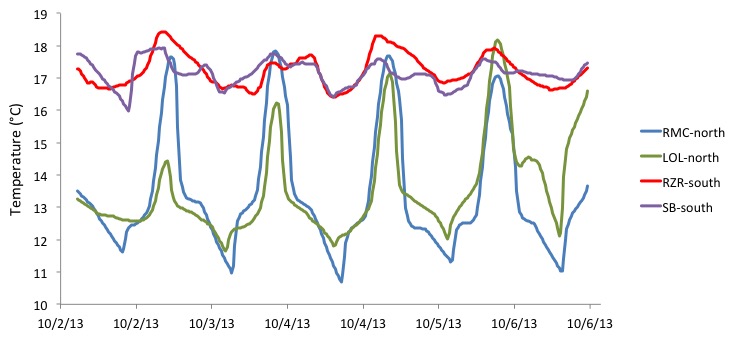

Supplement: Supplementary file 1 [file EVA-9-1043-s001.jpg]

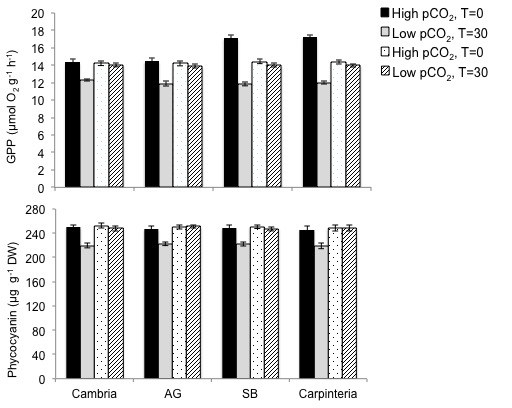

Supplement: Supplementary file 2 [file EVA-9-1043-s002.jpg]
